# Complete genome sequence of bacteriophage P8625, the first lytic phage that infects *Verrucomicrobia*

**DOI:** 10.1186/s40793-015-0091-0

**Published:** 2015-11-11

**Authors:** Ahyoung Choi, Ilnam Kang, Seung-Jo Yang, Jang-Cheon Cho

**Affiliations:** Department of Biological Sciences, Inha University, Incheon, 402-751 Republic of Korea

**Keywords:** Verrucophage, *Verrucomicrobia* bacteriophage, Genome, Marine phage, *Siphoviridae*

## Abstract

Bacteriophage P8625 is a lytic bacteriophage that infects the verrucomicrobial strain IMCC8625, a marine bacterium affiliated with *Verrucomicrobia* subdivision 4. Both the bacteriophage and the host bacterial strain were isolated from surface seawater samples collected off the east coast of Korea. The phage particle has an icosahedral capsid with a diameter of ~47 nm and a long tail of ~75 nm in length, showing the distinctive morphology of the *Siphoviridae* family. The complete genome sequence of phage P8625 is 32,894 bp long with 51.0 % G + C content. This is the first report of the complete genome sequence of a lytic phage that infects the *Verrucomicrobia*, for which the name “verrucophage” is proposed.

**Electronic supplementary material:**

The online version of this article (doi:10.1186/s40793-015-0091-0) contains supplementary material, which is available to authorized users.

## Introduction

Marine viruses are the most abundant biological components in the ocean, with ~10^7^ virus-like particles per milliliter in surface seawater [1–3]. Marine bacteriophages lyse specific bacterial hosts, controlling bacterial abundance and diversity and influencing biogeochemical cycles, which makes the study of marine viruses ecologically important [2, 4, 5]. Recent metagenomic studies have demonstrated immense genetic diversity among marine viruses [6–8]. However, these studies had difficulty in the phylogenetic interpretation of metagenomes due to the shortage of genetic data from representative marine viral isolates [7, 9]. Therefore, isolation of bacteriophages is also important for a better understanding of marine virome data [10, 11].

Bacterial members of the phylum *Verrucomicrobia* are distributed widely in the ocean environment, albeit in low densities [12]. Based on 16S rRNA gene sequence analyses, the phylum *Verrucomicrobia* was found to comprise an average of 2 % of the water column and 1.4 % of the sediment bacterial population [12]. Compared to marine environments, in terrestrial ecosystems such as soil environments, the phylum *Verrucomicrobia* is highly abundant and often exceed 20 % of the total rRNA gene sequences [13]. To our knowledge, however, no lytic phage infecting verrucomicrobial strains has been reported [14], perhaps largely due to the scarcity of bacterial hosts. Interestingly, a few recent studies based on fosmid sequencing or single cell genomics have hinted at the presence of verrucomicrobial phages in marine environments [15–17].

In this study, we report the isolation and genomic characterization of bacteriophage P8625, a novel marine siphovirus that infects the marine bacterial strain IMCC8625. Because the bacterial strain belongs to the class *Opitutae* [18] of the phylum *Verrucomicrobia*, phage P8625, isolated off the east coast of Korea, is regarded as the first isolated lytic phage of the *Verrucomicrobia*, for which the name “verrucophage” is proposed.

## Organism information

### Classification and features

Bacterial strain IMCC8625 was isolated using high-throughput cultivation based on dilution to extinction [19], during a survey of microbial assemblages inhabiting coastal seawater of the East Sea. A comparison of 16S rRNA gene sequences indicated that strain IMCC8625 was closely related to *Coraliomargarita akajimensis*DSM 45221^T^ (95.4 % similarity), a marine verrucomicrobial strain [20, 21]. Strain IMCC8625 was used as the bacterial host for the screening of marine lytic bacteriophages, which resulted in the isolation of verrucophage P8625.

**Fig. 1:**
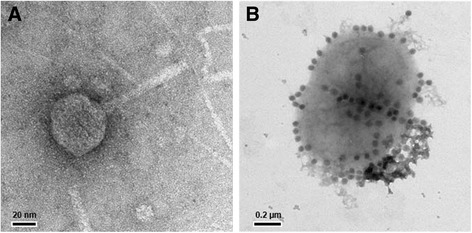
Transmission electron micrographs of verrucophage P8625 particles. **a** A single particle of P8625, **b** Bacteriophage particles attached to a host cell

Verrucophage P8625 was isolated from a surface seawater sample collected off the east coast of South Korea, in the East Sea (Sea of Japan). It is a lytic phage, forming plaques with a diameter of 1 to 2 mm after 5 days of infection of strain IMCC8625. Transmission electron microscopy of purified phage particles showed that P8625 had an icosahedral capsid approximately 47 nm in diameter and a long tail 71 ~ 75 nm in length (Fig. 1a). Bacteriophage P8625 attaches itself to and infects the host cell (Fig. 1b). The tail appears to be long and non-contractile, which is typically seen in members of the *Siphoviridae* family [22]. The genome of P8625 is comprised of dsDNA with a length of 32,894 bp and 51.0 % G + C content. The morphological evidence (Fig. 1), together with the dsDNA genome, has led to the tentative classification of P8625 as a member of the *Siphoviridae* family, with an unassigned genus. A summary of the isolation and general phylogenetic features of phage P8625 are shown in Table 1.

**Table 1:** Classification and general features of verrucophage P8625 according to the MIGS recommendation

MIGS ID	Property	Term	Evidence code^a^
	Classification	Domain: viruses, dsDNA viruses, no RNA stage	TAS [39]
		Phylum: unassigned	
		Class: unassigned	
		Order: *Caudovirales*	TAS [39]
		Family: *Siphoviridae*	TAS [39]
		Genus: unassigned	
		Species: unassigned	
		Strain: P8625	
	Particle shape	Icosahedral capsid with a long noncontractile tail	IDA
MIGS-6	Habitat	Marine surface water, coastal	IDA
MIGS-15	Biotic relationship	Intracellular parasite of *Verrucomicrobia* strain IMCC8625	IDA
MIGS-14	Pathogenicity	Lytic virus of strain IMCC8625	IDA
MIGS-4	Geographic location	East Sea (Sea of Japan), Sokcho, South Korea	IDA
MIGS-5	Sample collection	October 2, 2013	IDA
MIGS-4.1	Latitude	38° 14' 12'' N	IDA
MIGS-4.2	Longitude	128° 40' 59'' E	IDA
MIGS-4.4	Altitude	-	IDA

^a^Evidence codes - IDA: Inferred from Direct Assay; TAS: Traceable Author Statement. The evidence codes are from the Gene Ontology project [40]

## Genome sequencing information

### Genome project history

Bacteriophage P8625 was selected for genome sequencing because of its importance to understand bacteriophages infecting members of the *Verrucomicrobia* that are known to play crucial ecological roles in the ocean [23]. This bacteriophage that was shown to infect bacterium IMCC8625 in this study is the first lytic phage infecting *Verrucomicrobia*. This genome sequence has been submitted to GenBank, and the project information is also available in GOLD. A summary of the project information is shown in Table 2.

**Table 2:** Project information

MIGS ID	Property	Term
MIGS-31	Finishing quality	Finished
MIGS-28	Libraries used	One paired-end Illumina library
MIGS-29	Sequencing platforms	Illumina MiSeq
MIGS-31.2	Fold coverage	3085 ×
MIGS-30	Assemblers	SPAdes version 3.1.1
MIGS-32	Gene calling method	RAST version 2.0, GeneMark.hmm, and GLIMMER
	GenBank ID	KP792622
	GenBank Date of Release	April 5, 2015
	GOLD ID	Gp0111340
	BIOPROJECT	NA^a^
MIGS-13	Source Material Identifier	NA^a^
	Project relevance	Diversity of marine bacteriophage

^a^Not available

### Growth conditions and genomic DNA preparation

Verrucophage P8625 was isolated from a surface seawater sample collected at the station where the host strain IMCC8625 had been isolated, using the standard plaque assay after being enriched with the host. For the enrichment, the seawater sample was filtered using a 0.22-μm polyethersulfone membrane filter (Durapore, Millipore) to remove bacterial particles. To 400 ml filtered seawater, 100 ml of 5× R2A broth (BD Difco) and 30 ml of exponentially grown IMCC8625 culture were added. During incubation of this enrichment culture at 20 °C for 2 weeks, 10 ml of the culture was removed and treated with 2 ml of chloroform at 3 days interval. After centrifugation of chloroform-treated cultures to remove bacterial debris, bacteria-free supernatants were used for double agar overlay plaque assay. From an isolated plaque on the final assay plate, a single strain of phage was established and designated P8625.

The purification of phage DNA followed the method outlined in Molecular Cloning: A Laboratory Manual [24] with minor modifications. Approximately 2 l of phage lysates were prepared for DNA purification. DNase I and RNase A were added at a final concentration of 1 μg per ml. Then, 116.9 g of NaCl was dissolved in the lysates and cooled at 4 °C. After about 1 h, the mixed lysates were centrifuged at 10,000 × g for 30 min at 4 °C to remove the debris. Phage particles in the supernatant were precipitated with 10 % (*w/v*) PEG 8000 (Sigma-Aldrich). After an overnight incubation at 4 °C, the mixture was pelleted at 10,000 × g for 25 min at 4 °C and the pellet was gently resuspended in 2 ml SM buffer (50 mM Tris–HCl, pH 7.5; 100 mM NaCl; 10 mM MgSO_4_ · 7H_2_O; 0.01 % gelatin). PEG was extracted by treatment with an equal volume of chloroform. The phages were then ultracentrifuged at 246,000 × g for 2 h at 4 °C in an L-90 K ultracentrifuge (Beckman) with an SW 55 Ti rotor. Pelleted phage particles were resuspended with 100 μl of SM buffer overnight at 4 °C, and the purified phages were stored in the dark at 4 °C. The genomic DNA of P8625 was extracted using a silica based spin column (Qiagen DNeasy Blood and Tissue Kit) according to the manufacturer’s instructions.

### Genome sequencing and assembly

The genomic DNA of P8625 was sequenced at ChunLab, Inc. using an Illumina Miseq system with 2 × 300 bp paired-end reads. Assembly of the resulting reads was performed using SPAdes version 3.1.1 [25]. The Illumina platform provided 3085 × fold coverage of the genome. The genome was assembled into one contig through PCR-based gap closing.

### Genome annotation

The prediction of genes in the genome was performed using a combination of three gene calling methods: the RAST server [26], Genemark.hmm 3.25 [27], and GLIMMER version 3.02 [28]. Assignment of protein function to ORFs was performed manually using BLASTp against the NCBI nonredundant database and RPS-BLAST or HMMER search against the COG database [29], Pfam database [30], and TIGRFam database [31]. InterProScan was also used [32]. Search results were combined to assign a putative function for each predicted protein. TMHMM [33] and SignalP [34] were used to predict transmembrane helices and signal peptides, respectively.

## Genome properties

**Fig. 2:**
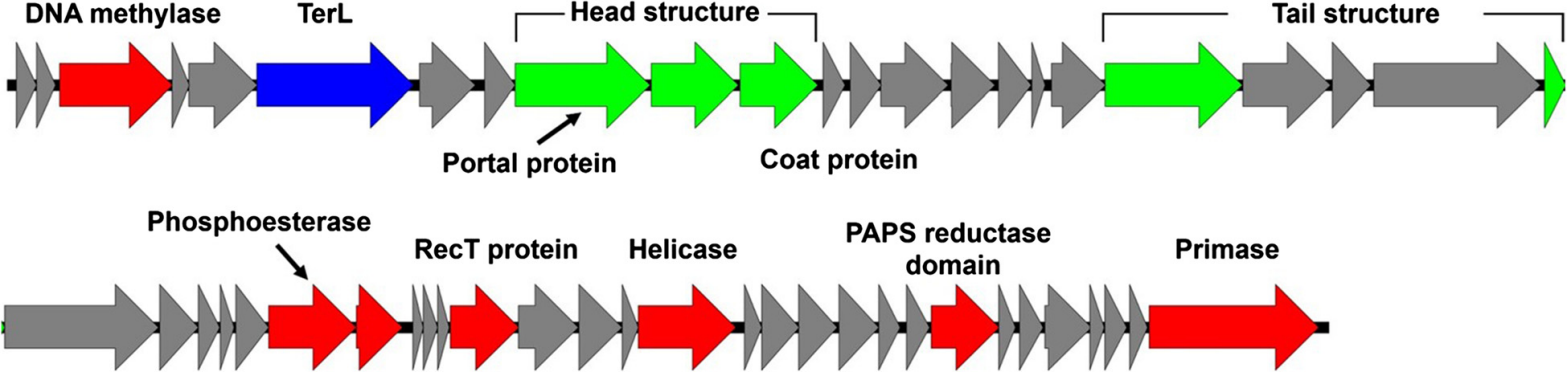
Genome map of verrucophage P8625. Total length of the genome is 32,894 bp and functional modules are indicated by color (Green: structure; Blue: DNA packaging; Red: DNA replication and metabolism; Gray: hypothetical proteins)

The properties and statistics of the genome are summarized in Table 3. The genome of P8625 was assembled as a 32,894 bp dsDNA, with a G + C content of 51.0 %. In total, 52 putative CDSs were predicted in the phage genome (Fig. 2). Of the 52 predicted protein-coding genes, 13 genes were assigned putative functions, whereas the remaining genes were annotated as coding for hypothetical proteins. Four proteins with transmembrane helices were identified, but signal peptides were not detected in any protein. The distribution of genes into COG functional categories is presented in Table 4.

**Table 3:** Genome statistics

Attribute	Value	% of Total^a^
Genome size (bp)	32,894	100.00
DNA coding (bp)	31,525	95.84
DNA G + C (bp)	16,765	50.97
DNA scaffolds	1	100.00
Total genes	52	100.00
Protein coding genes	52	100.00
RNA genes	0	0.00
Pseudo genes	0	0.00
Genes in internal clusters	0	0.00
Genes with function prediction	13	25.00
Genes assigned to COGs	8	15.38
Genes with Pfam domains	13	25.00
Genes with signal peptides	0	0.00
Genes with transmembrane helices	4	7.69
CRISPR repeats	0	0.00

^a^The total is based on the total number of protein-coding genes in the annotated genome

**Table 4:** Number of genes associated with general COG functional categories

Code	Value	% of Total^a^	Description
J	0	0.00	Translation, ribosomal structure and biogenesis
A	0	0.00	RNA processing and modification
K	0	0.00	Transcription
L	2	3.85	Replication, recombination and repair
B	0	0.00	Chromatin structure and dynamics
D	0	0.00	Cell cycle control, cell division, and chromosome partitioning
V	0	0.00	Defense mechanisms
T	0	0.00	Signal transduction mechanisms
M	0	0.00	Cell wall/membrane biogenesis
N	0	0.00	Cell motility
U	0	0.00	Intracellular trafficking and secretion
O	1	1.92	Posttranslational modification, protein turnover, and chaperones
C	0	0.00	Energy production and conversion
G	0	0.00	Carbohydrate transport and metabolism
E	1	1.92	Amino acid transport and metabolism
F	0	0.00	Nucleotide transport and metabolism
H	0	0.00	Coenzyme transport and metabolism
I	0	0.00	Lipid transport and metabolism
P	0	0.00	Inorganic ion transport and metabolism
Q	0	0.00	Secondary metabolites biosynthesis, transport and catabolism
R	0	0.00	General function prediction only
S	0	0.00	Function unknown
X	2	3.85	Phage terminase and bacteriophage capsid protein
-	44	84.62	Not in COGs

^a^The total is based on the total number of protein coding genes in the genome

## Insights from the genome sequence

When all 52 CDSs predicted from the P8625 genome were subjected to functional annotation based mainly on a conserved domain/motif search, only 13 CDSs were found to have specific functions. These functions were related to head and tail assembly, DNA packaging, or DNA replication and metabolism (Fig. 2 and Additional file 1: Table S1). Head and tail structural proteins were similar to those of the *Siphoviridae* family, while some proteins involved in DNA packaging and DNA replication/metabolism were similar to those of the *Myoviridae* family, suggesting mosaicism of the genome. The genome contained a putative RecT protein (CDS34) and a protein containing a PAPS reductase domain (CDS45) (Additional file 1: Table S1 and Fig. 2). RecT protein is involved in recombination repair of DNA [35], and homologs of RecT have been found in many phages [36]. The function of the protein containing the PAPS reductase domain (CDS45) is unclear. Although we cannot exclude the possibility of this protein being involved in sulfate metabolism, the location of this protein in the genome (Fig. 2) and the affiliation of PAPS reductase domain with the adenine nucleotide alpha hydrolase superfamily (cl00292) suggest that this protein could play a role in DNA metabolism.

**Fig. 3:**
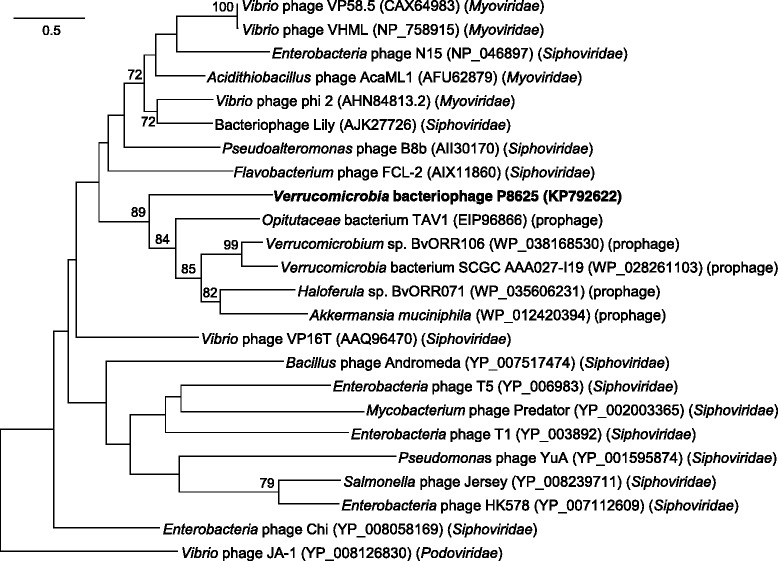
Phylogenetic tree showing the relationship of verrucophage P8625 (shown in bold) and other phages or prophages. The tree is based on aligned sequences of the TerL, using the *Vibrio* phage JA-1 as an outgroup. All sequences, other than P8625, were collected from NCBI and aligned using CLUSTALW and their evolutionary relationships were inferred through the maximum likelihood method using MEGA6. The bootstrap consensus was set at 1000 replicates, and bootstrap values (>70 %) are shown at branch nodes. Bar, 0.5 substitutions per amino acid position

A TerL, a commonly-used viral phylogenetic marker [37, 38], was predicted in the phage P8625 genome and used for phylogenetic analyses together with other representative tailed phages and *Verrucomicrobia* prophages (Fig. 3). In the resulting phylogenetic tree, the terminases from P8625 and *Verrucomicrobia* prophages formed a robust cluster supported by high bootstrap values (Fig. 3), which suggested that P8625 has a strong relationship with *Verrucomicrobia* prophages, without a clear affiliation with any of the three families of dsDNA tailed phages.

Several proteins predicted from the P8625 genome, including DNA methylase (CDS3), TerL (CDS6), and head structure proteins (CDS9–11), were similar to proteins annotated from a short (~14 kb) contig of a verrucomicrobial single cell genome SCGC AAA027-I19. This contig might have originated from a prophage in the host genome or might have been derived from a lytic phage during an infection cycle when the host cell was selected for multiple displacement amplification.

## Conclusions

The virulent verrucophage P8625, a siphovirus isolated from seawater in the East Sea, represents the first identification of a *Verrucomicrobia* phage. The dsDNA genome of P8625 was 32,894 bp long, with a 51.0 % G + C content. Among the 52 protein-encoding genes predicted in the genome, only 13 genes were functionally annotated, while the remaining 75 % of the genes were annotated as encoding hypothetical proteins. Verrucophage P8625 and its genome sequence can improve our understanding of the interaction of marine verrucomicrobial strains and their phages.

## Additional file

Additional file 1: Table S1. Verrucophage P8625 gene annotation. (PDF 77 kb)

## Abbreviations

PEG: Polyethylene glycol
PAPS: Phosphoadenosine phosphosulphate
TerL: Terminase large subunit
