# Supplementary material for: Complete genome sequence of bacteriophage P8625, the first lytic phage that infects Verrucomicrobia
Source: Stand Genomic Sci. 2015 Nov 11;10:96. doi: 10.1186/s40793-015-0091-0 (PMC4642752; doi:10.1186/s40793-015-0091-0)
Supplement: Additional file 1: Table S1. — Verrucophage P8625 gene annotation. (PDF 77 kb) [file 40793_2015_91_MOESM1_ESM.pdf]

Table S1. Verrucophage P8625 gene annotation

ORF	Strand	Size, aa	Predicted function	Most significant hit in BLASTP against nr (Organism, GenBank accession no., e-value)[1]	Domain or family (function, e-value)[2]
1	+	78	Hypothetical protein	-	-
2	+	72	Hypothetical protein	Caspase catalytic subunit p20 (*Beggiatoa* sp. PS, EDN69980.1, 9.8E-02)	-
3	+	429	DNA methylase	DNA methylase (*Psychrobacter* phage pOW20-A, YP_007673350.1, 2.0E-94)	COG0863 (DNA modification methylase, 7.4E-30)
4	+	64	Hypothetical protein	-	-
5	+	259	Hypothetical protein	-	-
6	+	595	Terminase large subunit	Hypothetical protein (*Verrucomicrobium* sp. BvORR106, WP_038168530.1, 2.0E-40)	COG5525 (Phage terminase large subunit, 2.7E-107)
7	+	213	Hypothetical prophage protein	Hypothetical protein (*Bacillus azotoformans*, WP_035193196.1, 1.0E-37)	PF13395 (HNH endonuclease, 1.4E-5)
8	+	117	Hypothetical protein	-	-
9	+	512	Phage portal protein	Bacteriophage capsid protein-like protein (*Akkermansia muciniphila* CAG:154, WP_022198864.1, 1.0E-42)	PF05136 (Phage portal protein, 2.8E-83)
10	+	330	Clp protease	Clp protease family protein (*Escherichia coli* 2-005-03_S3_C3, KDW63908.1, 4.0E-55)	COG0740 (Protease subunit of ATP-dependent Clp proteases, 7.5E-26)
11	+	295	P22_Coat Protein	Hypothetical protein (*Verrucomicrobium* sp. BvORR106, WP_038168543.1, 3.0E-42)	PF11651 (P22 coat protein - gene protein 5, 1E-55)
12	+	82	Hypothetical protein	-	-
13	+	118	Hypothetical protein	-	-

14	+	256	Hypothetical protein	-	-
15	+	167	Hypothetical protein	-	-
16	+	124	Hypothetical protein	-	-
17	+	48	Hypothetical protein	-	-
18	+	206	Hypothetical protein	-	-
19	+	521	Phage tail protein	Hypothetical protein (*Opitutaceae* bacterium TAV5, WP_009514341.1, 6.0E-18)	PF10145 (Phage-related minor tail protein, 5.1E-5)
20	+	334	Hypothetical protein	-	-
21	+	145	Hypothetical protein	-	-
22	+	634	Hypothetical protein	Hep_Hag repeat-containing protein (*Paenibacillus curdlanolyticus* YK9, EFM09636.1, 7E-24)	-
23	+	82	Tail fiber protein	Tail fiber protein (*Rhodobacter* phage RcapNL, YP_007518411.1, 3.0E-09)	-
24	+	584	Hypothetical protein	Glucose/sorbosone dehydrogenase (uncultured bacterium lac193, AHN98026.1, 7.0E-04)	PF13385 (Concanavalin A-like lectin/glucanases superfamily, 3.4E-05)
25	+	145	Hypothetical protein	-	-
26	+	81	Hypothetical protein	-	-
27	+	53	Hypothetical protein	-	-
28	+	124	Hypothetical protein	Hypothetical protein (*Verrucomicrobia* bacterium SCGC AAA164-I21, WP_020034972.1, 8E-26)	-

29	+	334	Phosphoesterase	Metallophosphoesterase (*Hydrogenobacter thermophilus*, WP_012963179.1, 9E-56)	COG0622 (Phosphoesterase, 3.0E-17)
30	+	175	Shikimate kinase	Adenylate kinase (*Xanthomonas axonopodis* pv. Phaseoli, KGK67072.1, 5E-31)	COG0703 (Shikimate kinase, 8.4E-14)
31	+	40	Hypothetical protein	-	-
32	+	54	Hypothetical protein	-	-
33	+	42	Hypothetical protein	-	-
34	+	260	RecT protein	Hypothetical protein (*Thermus* sp. 2.9, WP_039455637.1, 1.0E-29)	PF03837 (RecT family, 4.8E-13)
35	+	227	Hypothetical protein	Hypothetical protein (*Diplosphaera colitermitum*, WP_043588465.1, 7.0E-17)	-
36	+	164	Hypothetical protein	-	-
37	+	61	Hypothetical protein	-	-
38	+	370	DNA helicase	Helicase (*Stigmatella aurantiaca*, WP_002613094.1, 1E-22)	PF00270 (DEAD/DEAH box helicase, 2.3E-19)
39	+	67	Hypothetical protein	-	-
40	+	140	Hypothetical protein	Hypothetical protein (*Kytococcus sedentarius*, WP_012802107.1, 9.0E-04)	-
41	+	146	Hypothetical protein	-	-
42	+	152	Hypothetical protein	Hypothetical protein (Bacteria, WP_019977657.1, 9.0E-32)	-
43	+	72	Hypothetical protein	-	-

44	+	85	Hypothetical protein	-	-
45	+	257	PAPS reductase	Phosphoadenosine phosphosulfate reductase (*Peptoniphilus* sp. oral taxon 375, WP_009430476.1, 5.0E-61)	PF01507 (Phosphoadenosine phosphosulfate reductasefamily, 8.7E-33)
46	+	62	Hypothetical protein	-	-
47	+	89	Hypothetical protein	-	-
48	+	178	Hypothetical phage protein	Phage M3 protein (*Pseudomonas taiwanensis*, WP_031324622.1, 9.0E-08)	COG0817 (Holliday junction resolvasome/endonuclease subunit, 5.5E-43)
49	+	54	Hypothetical protein	-	-
50	+	83	Hypothetical protein	-	-
51	+	68	Hypothetical protein	-	-
52	+	644	DNA primase	DNA/RNA helicase (*Pseudomonas* sp. WCS358, WP_033701413.1, 2.0E-31)	COG0358 (DNA primase, 4.8E-35)

[1] Best BLASTP match from NCBI nr database (e-value $\leq 10^{-4}$).

[2] From search results of COG, Pfam and TIGRFAM, the one with most significant e-value ($\leq 10^{-4}$) was presented.
